# The problem of low variance voxels in statistical parametric mapping; a new hat avoids a ‘haircut’

**DOI:** 10.1016/j.neuroimage.2011.10.027

**Published:** 2012-02-01

**Authors:** Gerard R. Ridgway, Vladimir Litvak, Guillaume Flandin, Karl J. Friston, Will D. Penny

**Affiliations:** a Wellcome Trust Centre for Neuroimaging, UCL Institute of Neurology, London, UK; b Dementia Research Centre, UCL Institute of Neurology, London, UK

**Keywords:** EEG, electroencephalography, fMRI, functional magnetic resonance imaging, FWHM, full-width at half-maximum, GM, grey matter, MEG, magnetoencephalography, MIP, maximum intensity projection, MNI, Montreal Neurological Institute, ResMS, residual mean squares, SPM, statistical parametric mapping, PET, positron emission tomography, VBM, voxel-based morphometry, SPM, Low variance, VBM, MEG, EEG, Source reconstruction

## Abstract

Statistical parametric mapping (SPM) locates significant clusters based on a ratio of signal to noise (a ‘contrast’ of the parameters divided by its standard error) meaning that very low noise regions, for example outside the brain, can attain artefactually high statistical values. Similarly, the commonly applied preprocessing step of Gaussian spatial smoothing can shift the peak statistical significance away from the peak of the contrast and towards regions of lower variance. These problems have previously been identified in positron emission tomography (PET) (Reimold et al., 2006) and voxel-based morphometry (VBM) (Acosta-Cabronero et al., 2008), but can also appear in functional magnetic resonance imaging (fMRI) studies. Additionally, for source-reconstructed magneto- and electro-encephalography (M/EEG), the problems are particularly severe because sparsity-favouring priors constrain meaningfully large signal *and variance* to a small set of compactly supported regions within the brain. (Acosta-Cabronero et al., 2008) suggested adding noise to background voxels (the ‘haircut’), effectively increasing their noise variance, but at the cost of contaminating neighbouring regions with the added noise once smoothed. Following theory and simulations, we propose to modify – directly and solely – the noise variance *estimate*, and investigate this solution on real imaging data from a range of modalities.

## Highlights

► Statistical parametric mapping judges significance with a signal-to-noise ratio. ► Low noise, e.g. outside the brain, can yield artefactually high statistical values. ► Spatial smoothing can shift peaks substantially towards regions of low variance. ► Source-reconstructed M/EEG data exhibits the problem particularly severely. ► The problem can be addressed by modifying the noise variance estimate.

## Introduction

The statistical parametric mapping (SPM) approach to the analysis of neuroimaging data rests upon the application of frequentist statistics to reject a null hypothesis at a particular voxel, local maximum or contiguous cluster (Chumbley and Friston, 2009; Friston et al., 1994). The null hypothesis, for example of no functional activation or of no group difference in activity or local tissue volume, is commonly tested with a t- or F-contrast of the parameters in a general linear model (Friston et al., 2007). The t-statistic is a signal-to-noise ratio; the significance of the estimated contrast of the parameters is judged with respect to its standard error, which is proportional to the estimated standard deviation of the noise in the model. The F-statistic is a ratio of explained to unexplained variance, which can also be expressed (see Implications for F-contrasts section) as a squared signal-to-noise ratio.

Employing a ratio of signal to noise is necessary because there is no principled parametric method to control the false positive rate when declaring the signal alone to be ‘large’. Worsley et al. (1992) pooled voxels to estimate a spatially stationary noise variance, however, the spatially non-stationary voxel-wise variance estimate proposed by Friston et al. (1991) has been found more appropriate. However, a consequence of each voxel having its own variance estimate is that rejected null hypotheses could relate to unusually low noise variance, as well as or even instead of noteworthy signal.

SPM is intended for smooth (and usually additionally *smoothed*) data, which interacts with this issue, since blurring regions of signal with neighbouring low-variance background regions can cause the significant area to spread into the background, and can shift the peak significance towards the low-variance regions, as observed by Reimold et al. (2006). This reduces the localisation accuracy of the topological features.

Reimold et al. (2006) proposed to address these localisation accuracy problems by returning to consider the underlying signal (the ‘contrast’ image) within the clusters detected by the conventional t-statistic based procedure. More precisely, significant clusters are grown to accommodate neighbouring voxels with similarly large signal, and the signal itself is visualised in colour-coded maps in place of the usual t-values.^1^ However, this modification clearly cannot protect against the unwanted detection of clusters with low signal in regions of even lower variance.

Reimold et al. (2006) considered positron emission tomography (PET) and simulated data. The problem is ameliorated to some extent for functional magnetic resonance imaging. fMRI data typically have lower inherent smoothness and lower applied smoothing (at least for first-level, within-subject, analysis), together with a higher background noise level. However, the problem is far more severe for source-reconstructed magneto- and electro-encephalography (M/EEG). Here, the ill-posed inverse problem requires prior knowledge about the form of the activity in a given task. A commonly used prior is that activation should be spatially sparse, for example with ‘multiple cortical sources with compact spatial support’ (Friston et al., 2008), which means that low activity – and correspondingly low variance – will be wide-spread even within the grey matter (GM).

For voxel-based morphometry (VBM), the same problem has already been discussed in the literature (Acosta-Cabronero et al., 2008; Bookstein, 2001). (Acosta-Cabronero et al., 2008) proposed that it could be corrected by adding background noise to low-probability voxels in the GM segments. Specifically, following probabilistic tissue classification (Ashburner and Friston, 2005), random noise uniformly distributed between 0 and 0.05 was added to voxels with GM probabilities below 0.05; an approach they termed the ‘Haircut’ due to its removal of significant voxels outside the skull. Acosta-Cabronero et al. (2008) argued ‘intuitively, the statistical effect of noise, with mean and standard deviation an order of magnitude lower than [the probabilities in voxels confidently segmented as GM], being smoothed into GM tissue, can be neglected.’ However, they also observed that such a low level of added noise meant that ‘the blobs were not completely restored to the glass brain’, which leaves open the question of whether a noise-level sufficient to solve the problem fully might have a non-negligible effect on voxels with substantial tissue probability.

The real purpose of adding noise to the data in the Haircut technique is to inflate the error variance *σ*^2^. Changing the data, however, has the unwanted side-effect of altering the estimated parameters and the estimated smoothness, as discussed later. We therefore propose a more incisive modification: that the error variance *estimate* ${\hat{\sigma }}^{2}$ (distinguished by the addition of a hat) be directly altered, without requiring any modification of the original data and hence preserving the signal. In brief, we simply add a small value to the estimated error variance. This has only an inconsequential effect in regions with non-trivial signal and variance, but can preclude large statistical values in regions of very low noise, and help to preserve the localisation accuracy of the statistical peaks. This approach is effective and easy to implement; however, it requires us to define what we mean by ‘a small value’. In what follows, we evaluate a simple procedure for determining this value automatically. First, we motivate our approach and derive a heuristic using simulated data, then we validate it using real VBM, MEG and fMRI data.

## Theory

The main equations related to the contrast *c* of the parameters *β* in a linear model of the data in *n*-vector *y* (at a particular voxel) with design matrix *X* (whose Moore–Penrose pseudoinverse is denoted *X*^+^) are:

(1) β^=X+y

(2) $$\hat{\varepsilon }=y-X\hat{\beta }=Ry\text{,}\;R=I-XX^{+}$$

(3) σ^2=ε^′ε^n−rankX=y′RytrR

(4) t=c′β^σ^c′X′X+c∝c′β^σ^.

Consider the hypothetical scenario of smoothing an infinitesimal ‘point source’ surrounded by zeros. Both the contrast in the estimated parameters $c^{\prime }\hat{\beta }=c^{\prime }X^{+}y$ and the residual images $\hat{\varepsilon }=Ry$ are linear in the data, so smoothing the data smooths both similarly. The estimated noise standard deviation required for the denominator of the t-statistic is the square root of the residual mean squares (ResMS) ${\hat{\sigma }}^{2}=\left(y^{\prime }Ry\right)/tr\left(R\right)$, which is nonlinear in the data, and might appear more complicated. For example, for Gaussian random field data, the estimated standard deviation image would relate to a square root of a Chi-square random field. However, note that because all the residual images have the same spatial profile, this profile is preserved by the squaring and square-rooting operations and by the summation in between them, suggesting that the smoothing will affect the numerator and denominator equally. Supporting this argument, simulations like those described below but with the noise standard deviation tending towards zero, indicate that shape and smoothness of $\hat{\sigma }$ matches that of $\hat{\beta }$ so that the t-map becomes flat and theoretically infinitely extended (see also Chumbley and Friston, 2009). It is therefore clear why low, but non-zero noise, surrounding signals in regions of higher noise, can give rise to the spreading of t-statistic peaks observed in the literature (Acosta-Cabronero et al., 2008; Reimold et al., 2006).

### Simulations

To illustrate the nature of the problem and some potential solutions, simple two-dimensional data corresponding to a one-sample *t*-test are simulated. The underlying signal is generated as a point source at the centre (pixel coordinates 20,20) of a 40 × 40 pixel image, distributed normally with mean 100 and standard deviation 100. A total of *n* = 12 images are simulated, so that the expected t-value of the underlying source is

(5) $$\frac{\beta }{\sigma \sqrt{1/n}}=\sqrt{n}\approx \text{3.464.}$$

The images containing the point source are smoothed with a 10 pixel full-width at half-maximum (FWHM) Gaussian kernel. Similarly smoothed Gaussian noise is added to produce the final data. Two different noise standard deviations are employed: 0.01 and 2; the signal is generated only once, remaining identical for each noise level. Note that because the underlying signal occurs only at one pixel prior to smoothing and that its standard deviation is 50 times higher than that of the high noise level, the high-noise data can also be viewed as an example of applying the Haircut technique of Acosta-Cabronero et al. (2008) to the low-noise data (strictly, the Haircut would not alter the signal pixel itself, but that effect here is trivial).

**Fig. 1:**
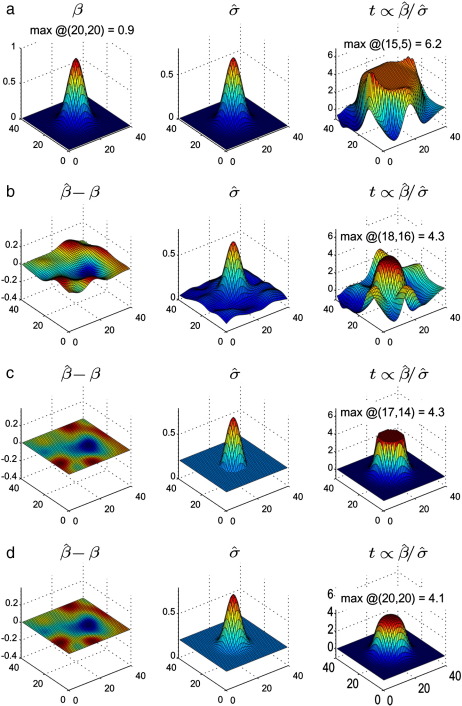
Simulation results. Columns, from left to right, show: the signal or its error (true signal *β* in first row; error in estimate $\hat{\beta }-\beta$ in rows b–d); the estimated noise standard deviation $\hat{\sigma }$; the SPM_*t*_. Rows correspond to: (a) Low-noise; (b) High-noise (or equivalently, added noise); (c) Low-noise with $\hat{\sigma }$ lower bounded at 0.2; (d) Low-noise with ${\hat{\sigma }}^{2}\to {\hat{\sigma }}^{2}+0.2^{2}$.

Fig. 1 (a) and (b) show results for the low- and high-noise data respectively. For the high-noise case, the estimated mean (beta) is very similar to the true value, so its discrepancy is plotted instead (the discrepancy for the low noise case matches that of rows c and d). For the low-noise case, as expected, the estimated noise standard deviation $\hat{\sigma }$ follows the same Gaussian shape as the signal, leading to a t-statistic map with a roughly flat plateau, surrounded by some more erratic values due to boundary effects. For the high-noise case, the t-map plateau is brought closer to the desired shape of the smoothed signal, though at the chosen noise level retains some distortion, with the maximal value displaced from the expected location by about 45% of the applied FWHM. Further increasing the noise level might improve the shape of the t-statistic surface, but at the expense of increasing the errors in the estimated parameter(s) *β*. There would also be an increasing risk that the non-zero sample mean of the noise itself could actually worsen the shape of the t-map, particularly with low degrees of freedom.

Alternative approaches are investigated in rows (c) and (d) of Fig. 1, each using the original low-noise data, but instead modifying the estimated noise standard deviation image. A reasonable lower bound for the (post-smoothing) noise standard deviation is 0.2, and in (c) the estimated standard deviation image is prevented from falling below this bound (values above the bound are left untouched). The original plateau is greatly reduced in diameter, and the Gaussian shape beyond the plateau made more similar to that of the signal. However, the discontinuity introduced to the $\hat{\sigma }$ image results in an undesirable discontinuity in the resultant t-map. Instead of a hard lower bound, therefore, in (d) we propose to modify the estimated noise standard deviation by adding the value of the bound. More precisely, we add the square of the bound to the estimated noise variance, in the expectation that this will impact less upon the pixels which already have suitably high $\hat{\sigma }$ due to the nonlinearity of the square root. That is, we propose a modification of the variance estimate,

(6) $${\hat{\sigma }}^{2}\to {\hat{\sigma }}^{2}+\delta \text{.}$$

Because the data (and hence the parameter estimates $\hat{\beta }$) are not altered, we are able to increase the level of $\hat{\sigma }$ beyond that which could be reasonably achieved with the Haircut technique, obtaining a satisfactory (though still slightly rounded) profile for the t-map, with a correctly located unique maximum value, and without any additional artefacts introduced away from the signal location. Although the addition of *δ* reduces the maximum t-value compared to using *δ* as a lower bound, the value of 4.1 is still above that of 3.46 expected for the underlying point source. This is due to the nonlinear effect on $\hat{\sigma }$ from smoothing lower noise regions together with the signal.

It appears that the addition of a small value *δ* to the estimated noise variance (known as the residual mean squares image in SPM and stored as ResMS.img) is an appealing strategy. However, in this illustration, the lower bound value was chosen by hand to produce satisfactory results. The problem of automatically choosing an appropriate value of *δ* for typical neuroimaging data is therefore addressed empirically in the following sections.

## Material and methods

Three separate modalities are explored: VBM, MEG and within-subject fMRI. In each case, the SPM software (version 8, revision 4290 — but without the modification that is described here) is used to estimate a general linear model and to compute a t-contrast of interest. To illustrate the potential problem at its most severe, the statistical modelling is performed at every non-constant voxel throughout the field of view, i.e. using no explicit mask and no threshold masking. SPM's implicit masking is still used along with the exclusion of voxels that are constant over all scans (which typically excludes only the voxels at the very edges of the field of view that are beyond the six-sigma support of the Gaussian smoothing kernel from any non-zero data). For the MEG data, the source reconstruction process means that the data are zero beyond a moderately tight grey matter mask.

The experimental approach is the same for each modality: the components of the t-statistic – the ‘contrast’ and the residual mean squares – are displayed; a histogram is used to estimate the distribution of the latter over voxels, and also a joint histogram of the contrast and ResMS, for reasons that will become clear in the results. Note that the joint histogram is only used to determine a suitable procedure from which *δ* can be estimated from the distribution of ResMS; the eventual (very simple) procedure is not dependent on a particular contrast (and thus can be enacted at the model-estimation stage without requiring any contrasts to be specified). The histograms employ the base-10 logarithm of ResMS; the fact that $\log_{10}\left({\hat{\sigma }}^{2}\right)=2\log_{10}\hat{\sigma }$ obviates the decision of whether to consider ResMS or its square-root. The original t-map is presented alongside the new version using the modified estimate of ResMS (${\hat{\sigma }}^{2}\to {\hat{\sigma }}^{2}+\delta$).

### VBM data

Structural MR images were obtained from the Open Access Series of Imaging Studies (OASIS) at http://www.oasis-brains.org/. We use the baseline scans from the longitudinal data-set (Marcus et al., 2010), which contains 150 subjects (62 males, 88 females) aged 60 to 96. 72 of the subjects were characterized as nondemented throughout the study, 64 were characterized as demented, and 14 subjects were characterized as nondemented at the time of their initial visit but were subsequently characterized as demented at a later visit.

Images were segmented using SPM8's New Segment toolbox (an extension of Ashburner and Friston, 2005) to produce native and ‘Dartel-imported’ (rigidly aligned to MNI orientation and resampled to 1.5 mm isotropic) segmentations of grey and white matter (WM). Dartel (Ashburner, 2007) was then used to nonlinearly warp all subjects together by simultaneously matching their GM and WM segments to an evolving estimate of their group-wise average (Ashburner and Friston, 2009). The transformations obtained (parameterised by flow-fields) were then applied to the native GM segments together with an affine transformation to MNI space. Probabilistic tissue volumes were preserved (‘modulation’). The images were finally smoothed with a Gaussian kernel of 8 mm FWHM.

All 150 subjects were modelled using SPM's flexible factorial design, with a three-level group factor (allowing for unequal variances). Covariates were included to adjust for age, gender and estimated total intracranial volume^2^ (Barnes et al., 2010). The contrast of interest tested for reduced GM in the 64 demented subjects compared to the 72 non-demented subjects.

### MEG data

We use the multimodal face-evoked responses dataset that is openly available from the SPM website, http://www.fil.ion.ucl.ac.uk/spm/data/mmfaces/, and is described in chapter 37 of the SPM Manual (SPM8 revision 4290). The data are for a single subject, undergoing the experimental paradigm developed by Henson et al. (2003) wherein subjects viewed faces and scrambled images of faces (using random phase permutation in Fourier space). The MEG data were acquired on a 275 channel CTF/VSM system, though one sensor was dropped due to a fault.

The first run (SPM_CTF_MEG_example_faces1_3D.ds) was processed; data were baseline-corrected with baseline between − 200 and 0 ms and downsampled to 200 Hz.

Multiple sparse priors (MSP) source reconstruction was performed, which uses a Variational Laplace procedure for automatic relevance determination (ARD), constructing an appropriately sparse solution by selecting from a large number of spatially compact putative sources (Friston et al., 2008).

Standard settings were used (Litvak et al., 2011), with the ‘MEG Local Spheres’ forward model (Huang et al., 1999), applied to the entire time series. The source power was summarised separately for each trial with a Gaussian window from 150 to 190 ms corresponding to the ‘M170’ peak in evoked response field (ERF). The power values were smoothed on the cortical mesh using 8 iterations of graph Laplacian smoothing, interpolated from the mesh to a regular three-dimensional volume using a non-linear interpolation method (spm_mesh_to_grid), and then smoothed in 3-D space with a 1 voxel FWHM Gaussian kernel.

A two-sample *t*-test was used to compare 20 trials with faces to 20 with scrambled faces.

### fMRI data

The fMRI time-series data is also a standard SPM data-set, available from http://www.fil.ion.ucl.ac.uk/spm/data/face_rep/ and described in chapter 29 of the SPM manual. It consists of a single session of data for one subject from a study of repetition priming for famous and nonfamous faces (Henson et al., 2002). The functional time-series comprises 351 volumes (repetition time 2 s) consisting of 24 descending slices (3 mm thick plus 1.5 mm gap; 64 × 64 matrix of 3 × 3 mm^2^) of echo planar imaging data (echo time 40 ms). A standard T1-weighted structural MRI is also available.

The data were preprocessed as described in the SPM manual: briefly, the volumes were realigned to correct for head motion, slice-timing discrepancies were corrected, the mean of the realigned functional time-series was coregistered to the structural image, the latter was segmented and the spatial transformation parameters from the unified segmentation (Ashburner and Friston, 2005) were used to spatially normalise the functional images, which were then smoothed with an 8 mm FWHM isotropic Gaussian kernel.

The data were modelled with two conditions, fame (famous face or not) and repetition (first or second presentation), in a 2 × 2 factorial design. The canonical haemodynamic response with time and dispersion derivatives were used to form regressors from the appropriate stimulus functions. Default values were used for other settings (128 s high-pass filter, serial correlations modelled as a first order autoregressive process).

The contrast of interest here is the positive effect of condition on the canonical terms, i.e. the activation in response to faces, averaged over the four cells of the factorial design.

## Results

**Fig. 2:**
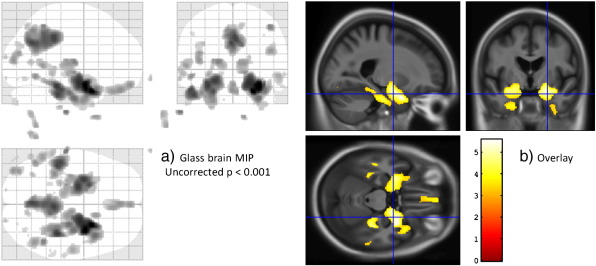
VBM results. Regions with reduced grey matter in subjects with dementia compared to controls, uncorrected *p* < 0.001. (a) ‘Glass brain’ maximum intensity projection (MIP) showing non-brain false positives (at this uncorrected level) due to low variance outside the brain. (b) Corresponding overlay of thresholded SPM_*t*_ on average template, with cross-hairs at the location of maximum ‘contrast’ value (effect or signal) rather than the more typically reported maximum t-value (signal-to-noise ratio) to provide context for Fig. 3.

**Fig. 3:**
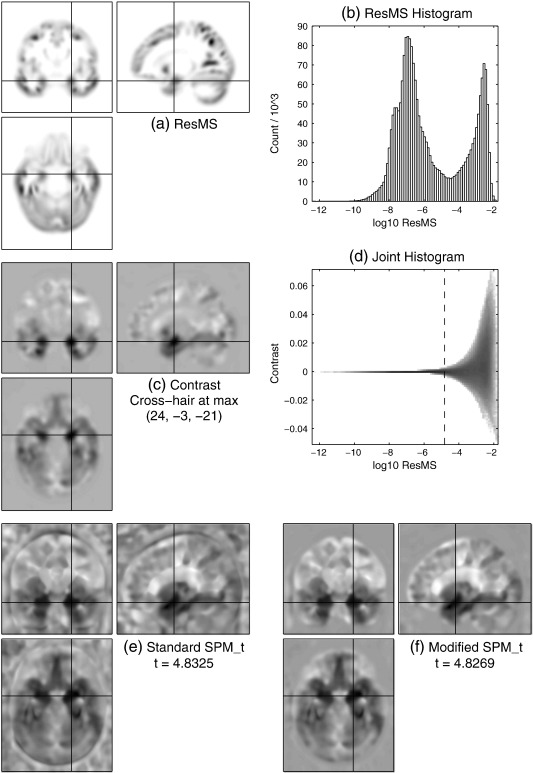
VBM data. (a) Image of estimated variance or residual mean squares (ResMS). (b) Histogram of *log*_10_ResMS over voxels. (c) The contrast of interest, labelled with the MNI coordinates of its maximum value. (d) Joint histogram of contrast value on the vertical axis and *log*_10_ResMS on the horizontal axis (which matches that of panel b). The vertical dotted line is located at *log*_10_*δ* = *max*(*log*_10_ResMS) − 3. (e) Statistical parametric map (SPM_*t*_) computed in standard way. (f) SPM_*t*_ computed using modified ResMS (with the amount shown dotted in panel d, *δ* = 10^− 3^ × *max*ResMS, added on). In panels a and d, white represents zero and higher values or counts are darker; in panels c, e and f, white values are negative and dark values are positive.

For the purpose of illustration, findings from the VBM data are shown as typically presented in Fig. 2; atrophy in the temporal lobes, posterior cingulate and precuneus is consistent with other reports (Baron et al., 2001; Lehmann et al., 2010), but non-brain regions are visible in the maximum intensity projection (MIP). Next, a series of figures investigating the relationship between the maps of ‘contrast’ (t-statistic numerator) and of estimated variance are presented, one for each modality studied. Figs. 2(b) and 3(a,c,e,f) show slices at the same location of maximal difference between estimated means for demented and non-demented subjects.

**Fig. 4:**
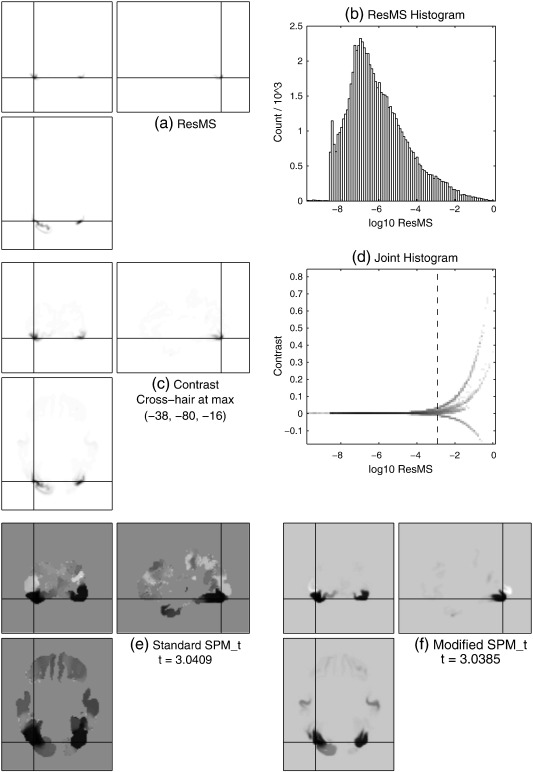
MEG data, following the same format as Fig. 3.

**Fig. 5:**
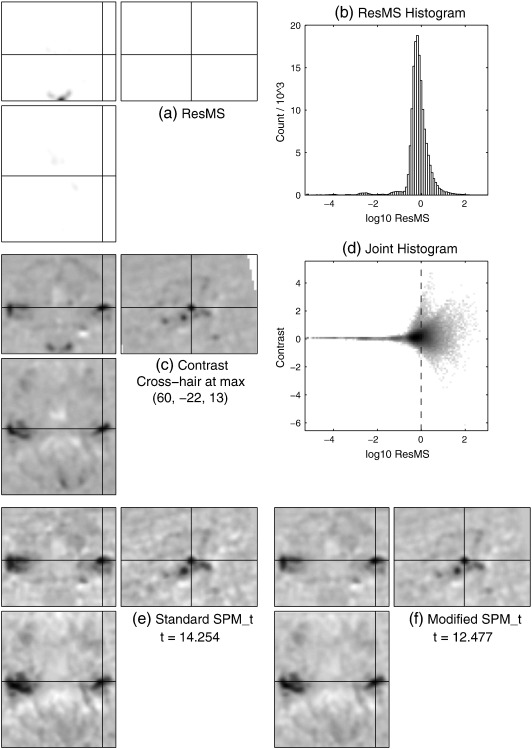
fMRI data, following the same format as Fig. 3.

For VBM data, the image of ResMS (Fig. 3 a) is very similar, in terms of the shape of its visibly non-zero regions, to the grey matter of the average template (Fig. 2 b), which reflects the fact that most of the variability (including the unexplained variability that relates to ${\hat{\sigma }}^{2}$) in VBM data is in regions with high GM probability. The corresponding panel (a) images for the MEG and fMRI data in Figs. 4 and 5 are strikingly different. For the MEG data, the ResMS image follows the pattern of the contrast image (Fig. 4 c) extremely closely, due to the nature of the MSP source reconstruction method. For the fMRI data, the ResMS is so much higher around the brain stem that its values over the grey matter are visually indistinguishable from the minimum value (white) when the intensity window is set to map the maximum value to black. Changing the intensity window (figure not shown) reveals a broadly uniform pattern over the brain, but with other outlyingly high values, including a very high spot visible near the centre of each slice due to a scanner artefact.

Considering the simple histograms, in Fig. 3(b), clear bimodality is visible, which appears to correspond to distinct foreground and background modes. In contrast, Fig. 4(b) is heavily skewed but shows no pronounced bimodality nor any clear distinction between foreground and background. Fig. 5(b) has a strong unimodal distribution, whose bulk is representative of the values found in grey matter, but with some quite severe outliers at both positive and negative extremes.

The dramatically varying distributions of the voxel-wise variance estimates makes it very difficult to find a suitable generic modelling strategy (attempts to fit mixtures of Gaussians with numbers of components selected by Bayesian model evidence proved unhelpful). This motivates consideration of the joint distribution of variance and signal estimates, in an attempt to find a heuristic background estimate.

The joint histogram for the MEG data (Fig. 4 d) reveals distinct curves; further investigation shows these correspond to separate clusters and are linear when contrast and $\hat{\sigma }$ are considered, thus they appear to represent the simple decay of signal and noise away from the centres of the compact MSP basis functions (see MEG data section). The other joint histograms show similar signal-to-noise relationships evident as the envelope of a much denser pattern, which arises due to the more complicated mixing of many more ‘sources’ in these modalities.

Based on visual inspection of these and other data-sets' joint histograms,^3^ the background estimate or lower bound *δ* to be added to the ResMS image was chosen to be one thousandth of the maximum value of the ResMS image. Correspondingly, the joint histograms are annotated with a vertical line at the value of

(7) $$\log_{10}\delta =\log_{10}\left(10^{-3}\times \mathrm{maxResMS}\right)=\max\left(\log_{10}\mathrm{ResMS}\right)-3.$$

This value seems appropriate for both VBM and MEG, but slightly too high for these fMRI data.

The efficacy of the simple modification for VBM and MEG can be seen by comparing the original and modified SPM_*t*_ images in Figs. 3 and 4 (e) and (f). For the VBM data, spurious non-brain findings have been dramatically reduced, and there is some evidence of slightly greater anatomical acuity within grey matter. The latter point is more clearly reinforced in the MEG results, where the original SPM_*t*_ without modification is unreasonably significant in several regions with very low signal and noise. Reassuringly, the t-values at the location of maximum signal are only reduced by about 0.1%, which should not change their statistical interpretation.

**Fig. 6:**
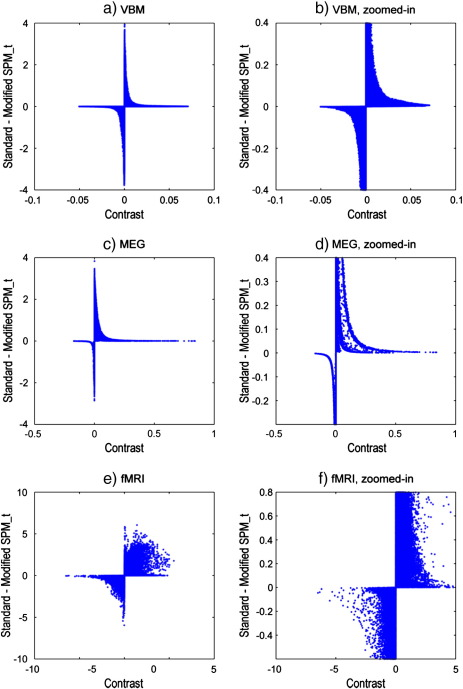
Comparison of t-statistic values from standard SPM_*t*_ and modified SPM_*t*_ using the altered ResMS. The difference in t-values (standard–modified) at each voxel is plotted against the contrast value at that voxel. The left column of plots show the full range of values, and the right column zooms in to enable values to be read off more accurately. (a,b) VBM. (c,d) MEG. (e,f) fMRI.

However, for the fMRI data, as could be expected from the location of *log*_10_*δ* on the joint histogram, the modification has had a more notable effect on the t-value at the location of maximum signal, which has been reduced by over 10%. The t-value changes are more precisely presented in Fig. 6, which plots the changes as a function of the underlying contrast value.

## Discussion

For VBM and MEG data, the problem of low-variance voxels is well addressed by the simple procedure of adding one thousandth of the maximum value to the residual mean squares variance estimate:

δ=10−3×maxσ^2σ^2→σ^2+δ.

Source-reconstructed EEG data is expected to behave similarly. However, for the fMRI data here, the reduction in the t-value at the location of maximal signal is over 10%, which is probably unacceptably high, in terms of the consequent reduction in power. This also seems to be true of other within-subject (first-level) fMRI data-sets, where artefacts are common but inconsistent in nature, leading to unpredictable behaviour of the joint distribution of signal and noise estimates. Furthermore, the problem of low variance voxels is itself less severe for within-subject fMRI, due to the smaller amount of smoothing typically applied and the strict masking procedure used by default at the first level.^4^

For between-subject (second-level) fMRI, the analysis mask is the intersection of all the first-level masks, which usually avoids including low-variance non-brain voxels.^5^ For these reasons, we do not recommend the procedure here for fMRI.

### Implications for F-contrasts

Although results have only been presented here for simple t-contrasts, the same low variance problem affects F-contrasts (with single or multiple column contrast matrices *C*), whose equivalent of Eq. (4) can be written in the form (Christensen, 2002, p. 69):

(8) $$F=\frac{{\left(C^{\prime }\hat{\beta }\right)}^{\prime }{\left(C^{\prime }{\left(X^{\prime }X\right)}^{+}C\right)}^{+}\left(C^{\prime }\hat{\beta }\right)}{{\hat{\sigma }}^{2}rank\left(C\right)}\text{,}$$

which shows that the same procedure of modifying ${\hat{\sigma }}^{2}$ while leaving $\hat{\beta }$ unaltered, also finesses the problem for F-contrasts.

### Relation to other procedures

As emphasised by Reimold et al. (2006), the numerator of the t-statistic or contrast image is important because it is probably the most spatially accurate way of locating effects (see also Poldrack et al., 2008 and http://imaging.mrc-cbu.cam.ac.uk/imaging/UnthresholdedEffectMaps). Nevertheless, thresholded SPM_*t*_ images remain the most common way of presenting results, and are more closely connected with the assessment of significance. Ultimately, the decision to use the approach of Reimold et al. (2006) or not depends on the particular research question, imaging modality and preferences of the investigator; the new method does not supplant careful consideration of the contrast image.

Due to its implementation prior to smoothing, the Haircut technique proposed by Acosta-Cabronero et al. (2008) can alter the contrast image in regions near to low probability areas (which arguably includes all of the cortex, if the smoothing FWHM is larger than the cortical thickness, as is usually the case), whereas our modification of only ResMS leaves the signal in the contrast image completely intact.

Overly generous masks might also be expected to influence estimation of the smoothness (Kiebel et al., 1999). The resels-per-voxel (RPV) image is typically very low away from the brain (the spatial gradients of low intensity regions tend to be very flat),meaning that the key quantity of interest – the resel count – is only weakly influenced by the inclusion of voxels with very low RPV. However, by adding initially rough noise to non-brain regions, the Haircut technique increases the RPV away from the brain, thus increasing the overall resel count and reducing the power of random field theory based inference. Our modification to ResMS has no impact on RPV estimation. 6

### Definition of the analysis mask

One might argue that the problem of low variance voxels can be solved simply by defining the analysis mask (Ridgway et al., 2009) to more strictly follow the grey matter, as for example in FSL-VBM (Douaud et al., 2007).^7^ This is perhaps partially true for fMRI and VBM, however, as noted by Acosta-Cabronero et al. (2008) and Ridgway et al. (2009), doing so can increase the risk that some true effects will be falsely excluded (particularly in morphometric studies of atrophied or damaged brains). Furthermore, the problem of SPM_*t*_ peaks shifting towards regions of lower variance – including those within the brain – would remain, whereas the modification of ResMS proposed here should ameliorate this problem (though admittedly not to the same extent as the method of Reimold et al., 2006). More importantly, with source reconstructed M/EEG data, even a very strict grey matter mask would include some regions of problematically low variance, and attempts to define a very strict signal-based mask might result in too few voxels for the reliable estimation of the smoothness needed for random field theory (Kiebel et al., 1999).

### Statistical shrinkage and Bayesian methods

The inflation of the estimated variance proposed here can also be viewed as a shrinkage of the estimated precision towards zero, motivating brief discussion of related statistical shrinkage procedures. The idea of shrinking or deliberately biassing an estimator to improve its performance with respect to some specified loss function was first proposed by Stein (1956) and extended by James and Stein (1961), though Tikhonov had worked on related concepts for integral equations and inverse problems in the 1930s and 40s (Kerimov, 2006). James and Stein (1961) showed that the obvious estimate for the mean of normally distributed samples (assumed to have unit variance for simplicity), which is the maximum likelihood and least-squares estimate, could be improved upon in terms of the estimator's expected squared error by shrinking it towards zero:

(9) $$\bar{y}\to \bar{y}-\frac{n-2}{{\bar{y}}^{\prime }\bar{y}}\bar{y}=\left(1-\frac{n-2}{{\bar{y}}^{\prime }\bar{y}}\right)\bar{y}\text{.}$$

With the goal of estimating covariance matrices, instead of mean vectors, Ledoit and Wolf (2004) derived a shrinkage estimator appropriate for high dimensional problems, which is the asymptotically optimal convex combination of the sample covariance matrix with a scaled identity matrix. This estimate has found application in neuroimaging as part of the ‘searchlight’ method of Kriegeskorte et al. (2006).

Another related application in imaging is wavelet shrinkage. Bullmore et al. (2004) review methods for denoising and for multi-scale spatial hypothesis testing using wavelet shrinkage, in which wavelet coefficients with absolute values below a threshold are zeroed and those above can be either preserved or have the threshold value subtracted from their absolute value, respectively known as ‘hard’ and ‘soft’ thresholding.

Bayesian statistics, in which one considers the posterior probability distribution of the aspect(s) of interest can also be used to derive shrinkage procedures. For example, considering *β* in a linear model to be a random variable having a Gaussian prior distribution with mean *μ*_*β*_ and covariance *Σ*_*β*_, the maximum a posteriori (MAP) estimate becomes a version of the ML estimate shrunk towards the prior mean, which generalises ‘ridge regression’ (Friston et al., 2002; Gelman et al., 2003):

(10) $${\hat{\beta }}_{\mathrm{MAP}}={\left(\sigma^{-2}X^{\prime }X+\sum {}_{\beta }^{-1}\right)}^{-1}\left(\sigma^{-2}X^{\prime }y+\sum {}_{\beta }^{-1}\mu \beta \right)$$

(11) $$={\left(X^{\prime }X+\sigma^{2}\sum {}_{\beta }^{-1}\right)}^{-1}\left(X^{\prime }X{\hat{\beta }}_{\mathrm{ML}}+\sigma^{2}\sum {}_{\beta }^{-1}\mu \beta \right)\text{.}$$

The form of the James–Stein estimator in (9) is clearly closely related to (11) with *μ*_*β*_ = 0 and *σ* = 1, except that in (9) ${\bar{y}}^{\prime }\bar{y}={\hat{\beta }}^{\prime }\hat{\beta }$ depends on the data, while in a conventional Bayesian setting the covariance *Σ*_*β*_ of the prior distribution would not. The notion of empirical Bayesian methods for hierarchical models allows the prior's hyper-parameters to be estimated from the data (Carlin and Louis, 2008; Friston et al., 2002), which can be shown to exactly generalise the James–Stein estimator (Lee, 2004). Similarly, wavelet shrinkage using soft thresholding can also be formulated as a Bayesian procedure with a sparsity-favouring prior over the wavelet coefficients, for example a Laplacian distribution (Bullmore et al., 2004) or a mixture of Gaussians (Flandin and Penny, 2007). Ledoit and Wolf (2004) also present a Bayesian interpretation of their estimator.

It is possible that an appropriate prior distribution for the variability *σ*^2^ could allow something similar to the modification of its estimate proposed here to be derived with an empirical Bayesian approach. This would have the advantage that the amount of modification could be derived from the data itself, instead of being arbitrarily set to some fraction (1/1000 here) of the maximum over voxels, which might conceivably allow the method to adapt more appropriately to fMRI data. However, this would require a somewhat different formulation than the usual hierarchical model in Friston et al. (2002), since the variance estimate becomes a parameter of interest in addition to $\hat{\beta }$, and is therefore left for further work.

Finally, on the topic of Bayesian methods, it is worth noting that the posterior probability mapping (PPM) approach (Friston and Penny, 2003) can entirely circumvent the problem of low-variance voxels undesirably becoming significant: instead of considering significance of each voxel in terms of the probability of the test statistic under the null hypothesis, the Bayesian approach can determine for each voxel the probability that the contrast of its parameters exceeds a specified effect size, and this effect size can be chosen to be non-trivial rather than simply non-zero. For an example of PPM applied to MEG data, see Henson et al. (2007).

### Conclusions

For modalities other than fMRI (specifically PET, structural MRI or VBM, and source-reconstructed EEG or MEG), we propose a conservative modification of SPM's residual mean squares image (ResMS) that simply entails adding on 0.1% of its maximum value.^8^ It has been shown here that the procedure has very limited effect on regions of meaningfully high signal, while avoiding the problem of artefactually high statistic values in regions with both low signal and low noise.

## Footnotes

1 Reimold et al.'s (2006) work resulted in an SPM toolbox for masked contrast images, MASCOI, http://homepages.uni-tuebingen.de/matthias.reimold/mascoi.

2 eTIV, obtained from http://www.oasis-brains.org/pdf/oasis-longitudinal.csv.

3 Two further VBM data-sets, two further fMRI data-sets, one further real and one simulated MEG data-set were similarly explored; results were broadly consistent with the exception of the fMRI data-sets, which differed with regard to the severity of their right-tail outliers.

4 The masking is not clearly documented, but in spm_fmri_spm_ui each image is thresholded at a fraction (by default 80%, defined in spm_defaults) of its global value estimated by spm_global (the mean of those voxels above one eighth of the original mean), and the intersection of all images' suprathreshold sets defines the overall analysis mask.

5 If one instead performed the first-level analysis in each subject's native space and normalised the contrast images, then the problem could reappear (e.g. as discussed on the SPM mailing list https://www.jiscmail.ac.uk/cgi-bin/webadmin?A2=SPM;ea0f015e.1012 and https://www.jiscmail.ac.uk/cgi-bin/webadmin?A2=SPM;715bcc4e.1012). This is also the reason why SPM's smoothing module has an option to preserve the implicit mask.

6 In more detail, spm_est_smoothness averages over voxels with ssq > sqrt(eps), where ssq is sum squared residual, which is equal to ResMS multiplied by the degrees of freedom, and sqrt(eps) is about 10^−8^. However, ssq is computed from the residual images themselves (stored temporarily in ResI_* files by spm_spm), not from the (potentially) modified ResMS image, so is unchanged by any modifications to the latter.

7 http://www.fmrib.ox.ac.uk/fsl/fslvbm/.

8 Software note: The modification has been released as part of revision 4290 (04-Apr-2011) of SPM8, available from http://www.fil.ion.ucl.ac.uk/spm/software/spm8/#Updates.
